# Supplementary material for: Wasted Children and Wasted Time: A Challenge to Meeting the Nutrition Sustainable Development Goals with a High Economic Impact to Ethiopia
Source: Nutrients. 2020 Nov 30;12(12):3698. doi: 10.3390/nu12123698 (PMC7760409; doi:10.3390/nu12123698)
Supplement: Supplementary file 1 [file nutrients-12-03698-s001.pdf]

nutrients

Supplementary Material

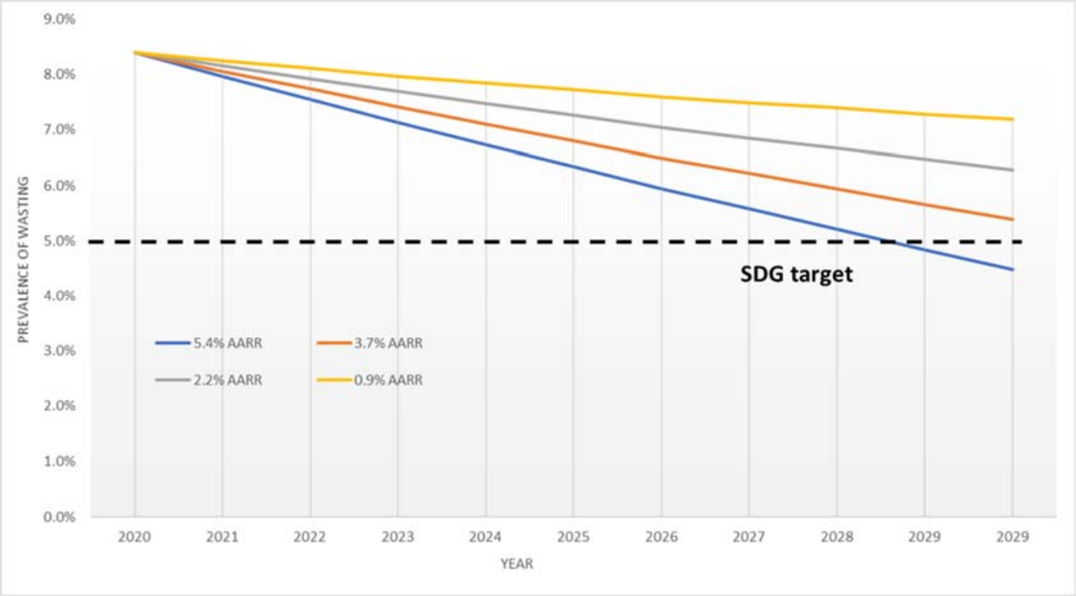

Figure S1. Trends of wasting prevalence from 2020 to 2030.

Publisher's Note: MDPI stays neutral with regard to jurisdictional claims in published maps and institutional affiliations.

© 2020 by the authors. Submitted for possible open access publication under the terms and conditions of the Creative Commons Attribution (CC BY) license (http://creativecommons.org/licenses/by/4.0/).
